# Ethnicity and Race Variations in Receipt of Surgery among Veterans with and without Depression

**DOI:** 10.1155/2011/370962

**Published:** 2011-10-12

**Authors:** Laurel A. Copeland, John E. Zeber, Mary Jo Pugh, Karon L. Phillips, Valerie A. Lawrence

**Affiliations:** ^1^Central Texas Veterans Health Care System, Research Service, Temple, TX 76502, USA; ^2^Center for Applied Health Research, Scott & White Healthcare, Temple, TX 76504, USA; ^3^VERDICT Research, South Texas Veterans Health Care System, 7400 Merton Minter (11c6), San Antonio, TX 78229, USA; ^4^Departments of Epidemiology & Biostatistics and of Medicine-Epidemiology, The University of Texas Health Science Center at San Antonio, San Antonio, TX 78229, USA

## Abstract

To examine equity in one aspect of care provision in the Veterans Health Administration, this study analyzed factors associated with receipt of coronary artery bypass graft (CABG), vascular, hip/knee, or digestive system surgeries during FY2006–2009. A random sample of patients (*N* = 317, 072) included 9% with depression, 17% African-American patients, 5% Hispanics, and 5% women. In the four-year followup, 18,334 patients (6%) experienced surgery: 3,109 hip/knee, 3,755 digestive, 1,899 CABG, and 11,330 vascular operations. Patients with preexisting depression were less likely to have surgery than nondepressed patients (4% versus 6%). In covariate-adjusted analyses, minority patients were slightly less likely to receive vascular operations compared to white patients (Hispanic OR = 0.88, *P* < .01; African-American OR = 0.93, *P* < .01) but more likely to undergo digestive system procedures. Some race-/ethnicity-related disparities of care for cardiovascular disease may persist for veterans using the VHA.

## 1. Introduction

Many studies have looked at the presence and effect of postoperative depression upon surgical recovery and other outcomes, but few have considered preoperative psychiatric status. A systematic review of the literature on postoperative clinical outcomes for patients with preexisting severe mental illness uncovered only 10 studies on patients with schizophrenia, two on patients with major depressive disorder, and none on patients with either bipolar disorder or posttraumatic stress disorder [1]. These four diagnoses comprise severe mental illnesses (SMI) affecting many aspects of life and health. The available reports suggested there may be worse outcomes for these patients including pulmonary thromboembolism and sepsis, potentially due to delayed presentation or management of acute disease [2, 3]. For depressed patients whose antidepressant medications are discontinued prior to surgery, worse postoperative depressive symptoms may ensue [4]. 

In addition, some reports have noted differences in the receipt of surgical interventions for ethnic minority patients [5–7] although others report no observable inequities, for example, in outcomes of colon cancer resection given equal access to care [8]. Equity in the provision of care is a primary goal of the Veterans Health Administration (VHA). The VHA treats a large, disadvantaged patient population selected from among US veterans of military service, including approximately 25–30% non-white veterans [9, 10], with uncertainty arising from missing data. 

Major depressive disorder (MDD) is the most commonly occurring mental illness in the United States, affecting up to 17% of persons by age 54 per epidemiological studies [11, 12]. In a large integrated health care system such as the VHA, one out of 11 patients has an SMI, suggesting that patients undergoing invasive surgery will often have severe psychiatric comorbidity. In addition, the VHA treats 150,000 patients for MDD annually. The VHA's system for monitoring surgical outcomes, Veterans Administration National Surgical Quality Improvement Program (VASQIP; formerly NSQIP), is a model of surgical quality improvement that has been adapted by the American College of Surgeons for use in nonfederal hospitals [13, 14]. Yet this comprehensive data collection and analysis program does not collect preoperative measures of psychiatric comorbidity, preventing its use to examine the question of parity in provision of surgery for patients with psychiatric disorders. In addition, the VASQIP sampling schema includes a cap on the number of procedures of any type assessed per month so that estimating prevalence of specific surgeries is not possible. The Surgical Treatment Outcomes of Patients with Psychiatric Disorders (STOPP) Project was funded by the VHA Health Services Research and Development program to address this methodological gap.

While VHA has made eliminating disparities in care a priority for some time [15], little work has been done in the area of surgical treatment. Data from VASQIP have provided evidence that African-American and Hispanic veterans were more likely to undergo amputation to treat peripheral artery disease, above the excess expected from higher rates of diabetes and hypertension in these groups [16]. Increased complications subsequent to knee (but not hip) surgeries have also been noted for African-American and Hispanic VHA patients compared to white patients [17].

Given the large number of veterans with psychiatric disorders, the complexity of managing cooccurring serious medical and mental disease, and the paucity of knowledge regarding surgery in patients with psychiatric disorders, the purpose of this paper was to examine race and ethnicity as factors potentially associated with surgeries experienced by patients with and without major depressive disorder.

## 2. Materials and Methods

The STOPP project assembled data from administrative extracts of the VHA's all-electronic medical records system for patients treated during the fiscal years 2005–2009 (fiscal year 2005 runs from October 1 2004 through September 30 2005). During this period, VHA treated approximately 7 million veterans, providing 78 million outpatient care visits and half a million inpatient stays per year nationwide. We identified procedures by CPT or ICD-9-A codes [18, 19]. Among surgeries, some of the most common were coronary artery bypass graft (CABG), vascular operations, hip and knee repairs and replacements, and surgeries on the digestive tract. Prior to the STOPP study, the research team conducted a 2-year pilot study (the POSSE project, also funded by VHA) to review and catalogue surgery procedure codes, selecting inpatient surgeries not performed solely for diagnostic purposes and categorizing all VHA patients treated in fiscal year 2005 as having SMI or not. This approach was then applied to VHA patients treated in FY2006–2009 for the STOPP project. For patients with more than one surgery date during 2006–2009, the first was selected and defined as the index surgery. 

### 2.1. Sample

Patients were eligible for inclusion if they were receiving care in the VHA in fiscal year 2006. Inclusion criteria were US military veteran status per priority score, valid race/ethnicity and gender data, valid date of birth, and logical mortality data (no record of a date of death prior to surgery date for the unique patient identifier). Because of the large number of patients treated each year (more than 5 million), a 10% random sample of those with nonmissing demographic data was taken. VHA outpatient data have high levels of missing data; patients with inpatient care or frequent visits are more likely to have valid race/ethnicity data [20]. Thus, patients included in the random sample were drawn from the 3.5 million patients with valid demographic measures. After identifying the cohort, we determined whether patients had any of the following nonambulatory surgeries during fiscal years 2006–2009: coronary artery bypass graft (CABG), vascular operations, hip or knee repairs and replacements, or surgeries on the digestive tract. Patients with other invasive surgeries as catalogued by the STOPP project were excluded for this report. The final sample size was 317,072. CABGs and vascular operations relate to disorders of the cardiovascular system, while the hip, knee, and digestive surgeries were selected as contrasting procedures. The codes defining our four selected surgery types are presented in Table 1.

### 2.2. Measures

The prior 12 months' data were used to determine depression status (depressed yes/no) and comorbidity burden at the time of surgery; for nonsurgery patients, fiscal year 2006 data were used. The Selim index of chronic physical conditions summed presence or absence of 30 comorbid conditions such as hypertension and irregular heartbeat based on diagnoses from the 12 month period [21]. The Selim was developed on patient self-reported conditions, validated against the Medical Outcomes Study Short Form Health Survey (SF-36), and later operationalized with VHA administrative data [22–24]. The Charlson comorbidity score summed 19 conditions weighted for their association with posthospitalization 1-year mortality. This measure was developed via chart review and later operationalized with ICD-9 codes from administrative data [25, 26]. Additional data aggregated from the inpatient and outpatient record extracts were age at surgery, gender, race (African-American, white, and other), and Hispanic ethnicity. Multiple records per patient were summarized to assign the most commonly reported race across multiple patient encounters, choosing self-report data over observer-reported values when available. Priority score ranges from 1 to 8 summarizing why a veteran is eligible for VHA care. Priority 1 veterans have 50–100% disability as a result of their military service and receive VHA care without copayments. Other categories include lower levels of service-connected disability, catastrophic disability (Priority 4), and low income (Priority 5). Priority is thus related to both socioeconomic status and severity of illness. 

Diagnosis with major depressive disorder (MDD) was determined by receipt of outpatient care on two or more different dates in one year for MDD as indicated by ICD-9 diagnosis codes 296.2, 296.3, or 311. The reliability of VHA administrative databases has been ascertained by many health services studies examining demographic characteristics, types of care received, and diagnoses, noting missing data on race/ethnicity as a common problem [27–30]. Diagnoses in administrative data are generally reliable when at least two dates of care list the same diagnosis [31–33].

### 2.3. Analysis

Patients were described by frequencies and means prior to modeling receipt of surgery as a function of clinical and demographic measures. Bivariate differences were assessed by chi-square analysis for categorical variables, by Student *t*-test for continuous measures when comparing two groups, and by analysis of variance for continuous measures by more than two groups (e.g., among the race categories). In the main models, multivariable logistic regression analyzed the relative odds of receiving a particular type of surgery as a function of race/ethnicity, age, gender, priority, depression status, and comorbidity burden (Selim Physical Comorbidity Index, Charlson Comorbidity Score). In exploratory models, we tested for an interaction between race or ethnicity and depression. An interaction asks whether the effect of one factor is changed when in the presence of another factor (has a synergistic or multiplicative relationship with the outcome). The types of surgery modeled as outcomes of the multivariable analyses were digestive system procedures, hip/knee procedures, CABG, and vascular operations. Results of the multivariable logistic regression models were reported as adjusted odds ratios (OR) with 95% confidence intervals. Odds ratios greater than 1.67 (i.e., a ratio of 4 : 3) or smaller than.  75 (3 : 4) suggest a medium effect, and those greater than 2 (2 : 1) or smaller than 0.5 (1 : 2) denote a large effect [34]. The values between 1 and 0 (fractional values) represent “protective” effects or inverse associations, while values greater than 1 represent risk factors or positive associations with receipt of the specific surgery modeled.

## 3. Results

Among the 317,072 patients sampled, 18,334 had surgery (6%). Patients in the study sample averaged 63 years of age (SD 15, range 18–103) and included 5% women veterans, 5% Hispanic, and 17% African-American patients. Hispanic ethnicity was assessed independently of race; thus 4,380 individuals were identified as both Hispanic and African-American (1.4% of the sample). Most patients were qualified for VHA care via low income (35%; Priority 5), while 1 in 6 (17%) qualified with high levels of service-connected disability (Priority 1). Among the four types of surgeries studied, 3,755 patients had surgeries of the digestive system, 3,109 had hip/knee procedures, 1,899 received CABG, and 11,330 had vascular operations; some patients (1,759) had more than one of these surgeries. Patients diagnosed with major depressive disorder numbered 27,296 (9%) including 998 undergoing one of the four inpatient surgeries (see Table 2). The proportions of all patients in the sample, Hispanic patients, and African-American patients with each of the chronic conditions identified by the Selim algorithm are shown in Table 3.

Hispanic patients were about 4 years younger than non-Hispanic patients on average (58.3 versus 62.9 years,  *t* = 35.8, df = 18388, *P* < .001), and African-American patients averaged 8 years younger than other patients (55.8 versus 64.1, *t* = 121.9, df = 79415, *P* < .001). Women, as might be expected from military recruitment patterns, were considerably younger than men by almost 15 years (48.7 versus 63.5 years, *t* = 111.7, df = 17766, *P* < .001). Patients with MDD were younger than their nondepressed counterparts (56.6 versus 63.3, *t* = 78.0, df = 33953, *P* < .001) and were less likely to undergo surgery than nondepressed patients (3.7% versus 6.0%, chi-square = 247.8, df = 1, *P* < .001). Hispanic patients were also somewhat less likely to have one of the four surgeries (5.2% versus 5.8%, chi-square = 10.1, df = 1, *P* < .01), but African-American race was not associated with a difference in frequency of these surgeries at the bivariate level (5.7% versus 5.8%, chi-square = 1.07, df = 1, *P* = .30 ns). 

Turning to the multivariable models of the primary outcomes of interest (receipt of specific types of surgery), in adjusted analyses Hispanic patients were more likely to get digestive surgeries (Odds Ratio [OR] = 1.3; 95% Confidence Interval 1.1–1.4) and somewhat less likely to have vascular operations (OR = 0.88; 0.81–0.97) relative to non-Hispanic white patients; no association with Hispanic ethnicity was noted for hip/knee procedures or CABG (see Table 4). African-American patients were slightly more likely to have digestive surgeries (OR = 1.1; 1.1–1.2) but significantly less likely to have CABG (OR = 0.56; 0.49–0.65) or vascular operations (OR =.93; 0.88–0.98) relative to white patients; no association with African-American status was noted for hip/knee procedures. 

In the exploratory models, no significant interactions between MDD and Hispanic ethnicity or between MDD and African-American race were observed. Thus, these factors had an additive but not multiplicative relationship to the outcome. 

Depressed patients were significantly less likely to have digestive procedures (OR = 0.46; 0.40–0.54), hip/knee procedures (OR = 0.49; 0.42–0.57), CABG (OR = 0.40; 0.32–0.50), or vascular operations (OR = 47; 0.43–0.51). In addition to the race/ethnicity and depression effects, a gender effect was noted: women were more likely to have digestive procedures (OR = 1.3; 1.1–1.5) and less likely to undergo CABG (OR = 0.29; 0.20–0.43) or vascular operations (OR = 0.51; 0.44–0.58). Comorbidity had mixed association with receipt of the different types of surgery: both chronic conditions (per Selim) and those related to posthospital mortality (per Charlson) were positively associated with digestive system surgeries, but having a higher Charlson score (higher risk of post-hospital death) was associated with reduced likelihood of hip/knee procedures. More comorbid chronic conditions per Selim correlated with increased relative odds of hip/knee procedures.

## 4. Discussion 

During the period 2006–2009, depression showed a pervasive dampening effect on the likelihood of having digestive, hip/knee, vascular, or CABG surgeries. Furthermore, Hispanic ethnicity and African-American race were inconsistently associated with decreased likelihood to get surgery in the VHA relative to non-Hispanic white patients. In fact, digestive surgeries were relatively more common among minority patients. At the same time, both coronary artery bypass graft and vascular operations were less common among minority veterans, even after controlling for mortality-associated comorbidity including several cardiovascular diagnoses, chronic conditions, age, gender, priority, and depression status. Given Hispanic and African-American patients somewhat younger age, some of this reduction in cardiovascular surgical care may be attributable to lack of need as both coronary and vascular diseases are age-related, although comorbidity (Selim, Charlson, and individual diagnoses) did not vary consistently across race/ethnicity groups; hypertension was as prevalent among African-American as white veterans for example (57%). While we present information on several potentially related comorbidities, the lack of information regarding severity of illness makes it difficult to determine whether or not diagnostic differences explain differences in surgery. 

The much greater disparity in CABG for African-American veterans is more challenging to understand. A prospective study by Hannan and colleagues found that after controlling for appropriateness and necessity for CABG surgery, which accounts for disease severity, African-Americans were still significantly less likely to receive CABG. Because the clinician recommended CABG only 10% of the time when an African-American patient did not receive an “appropriate” CABG, it appears that neither patient refusal nor disease severity accounted for this disparity. Castellanos and colleagues have investigated access to high-quality surgeons for minority patients in California's nonfederal (i.e., excluding VHA) hospitals, finding that on average Hispanic and African-American (as well as Asian) patients underwent CABG at the hands of surgeons with worse postoperative mortality ratings [35]. Possibly there is greater reluctance to risk surgery given publicity about findings of this nature. Exposure to California surgeons with worse ratings was most likely an issue of access to care, an issue which is reduced in the federal VHA. Previous research indicates that disparities within the VHA appear to be related to processes that involve either patient-provider communication issues or more effort on the part of patients/providers such as surgery and invasive procedures [15]. Nonmedical options, particularly prayer, seem to have greater value among African-Americans than among their white counterparts for managing conditions such as arthritis [36–38]. Similarly, African-American VA patients with bipolar disorder have demonstrated a greater willingness to use CAM as well [39].

Cram and colleagues reported lower rates of revascularization (percutaneous coronary intervention (PCI) or CABG) among African-American patients relative to white or Hispanic patients after adjusting for demographics, comorbidity, and insurance status but were unable to explain the residual differential [40]. A few years earlier, Becker and Rahimi documented excess in-hospital mortality risk for African-American or female CABG patients [41]. Our findings accord with the Cram and Becker studies, also finding a difference particular to African-American patients rather than Hispanic patients.

Presurgical major depressive disorder was uniformly associated with reduced likelihood of surgery, and this association showed a large effect (more than two-fold). Furthermore, the effect of depression was independent of race and ethnicity; thus, depression and African-American race would have an additive but not synergistic effect, each factor diminishing the depressed African-American patients' relative odds of CABG. Patients with MDD may be less likely to follow preoperative self-management guidelines, may choose nonsurgical therapies, or may be considered poor risks for surgery given the known association of postoperative depression with poor healing. For example, Doering and colleagues noted a positive association between depressive symptoms and objective evidence of impaired wound healing [42]. Alternatively, patients with major depression may have more negative views of surgery, as an extension of the hopelessness that characterizes depression [43]. In addition, some research has noted diminished benefit from CABG accruing to patients with postoperative depressive symptoms [44]; thus providers may believe that patients with depression are better off waiting for an improved mental status prior to revascularization surgery. Examining long-term outcomes could be helpful to illuminate the relative benefits of alleviating depression prior to revascularization weighed against the potential benefits of earlier revascularization. 

Because of historical recruitment trends in the armed services, women in the VHA are younger than men as well as underrepresented. Thus, there is a recognized interaction between gender and age. Due to the small numbers of women undergoing surgery, it was not possible to model this interaction explicitly, but it most likely accounts for the majority of the gender effect.

The sample studied in this paper was taken from VHA patients with valid data on race/ethnicity in administrative extracts from the VHA electronic medical records system. The sample was mostly male due to military recruitment patterns. Patients who visited the VHA less often or used only outpatient care were more likely to have missing data on race/ethnicity [20]. Thus, the study sample was biased toward sicker patients. Results may not generalize to healthier patient samples or women. Generally speaking, VHA patients are sicker than US residents [45, 46] and may more closely resemble Medicaid patients than patients with access to private insurance.

## 5. Conclusions

Our findings demonstrated some disparity with regard to race/ethnicity largely consistent with previous research. Moreover, our study demonstrated a strong effect of preoperative depression with depressed individuals being far less likely to receive these types of surgical procedures. In addition to exploring the combined influence of multiple vulnerabilities, such as chronic mental illness and ethnicity, with its accompanying potential disparities of care, future research examining variation in outcomes of surgery in preoperatively depressed patients is needed to determine the impact of this apparent disparity.

## Figures and Tables

**Table 1:** Definitions of four common types of surgery.

Surgery	Code schema	Values included
Coronary artery bypass graft (CABG)	CPT	33510, 33511, 33512, 33513, 33514, 33516, 33517, 33518, 33519, 33521, 33522, 33523, 33533, 33534, 33535, 33536, 92975, 92977, 92980, 92981, 92982, 92984, 92995
	ICD9A	3603, 3611, 3612, 3613, 3614, 3615, 3616, 3617, 3619, 363

Other vascular operations	CPT	33200, 33206, 33207, 33208, 33210, 33212, 33214, 33218, 33222, 33223, 33233, 33246, 33322, 33405, 33422, 34111, 34201, 34800, 34803, 34804, 34808, 34812, 34825, 34826, 35081, 35102, 35131, 35141, 35151, 35152, 35190, 35207, 35221, 35301, 35363, 35390, 35450, 35470, 35471, 35473, 35474, 35475, 35476, 35493, 35495, 35509, 35456, 35556, 35558, 35566, 35571, 35582, 35646, 35656, 35661, 35840, 35860, 35879, 35881, 36818, 36819, 36821, 37720, 37730, 37785, 37799
	ICD9A	00.50, 00.51, 00.56, 00.57, 00.58, 00.59, 00.60, 00.62, 00.66, 00.67, 00.68, 00.69, 35.0, 35.11, 35.12, 35.13, 35.14, 35.21, 35.22, 35.23, 35.24, 35.27, 35.28, 35.33, 35.39, 35.4, 35.50, 35.51, 35.53, 35.54, 35.55, 35.56, 35.57, 35.58, 35.59, 35.61, 35.62, 35.71, 35.72, 35.8, 35.91, 35.93, 35.95, 35.99, 36.01, 36.02, 36.04, 36.05, 36.06, 36.07, 36.09, 36.32, 36.39, 36.91, 36.99, 37.10, 37.11, 37.12, 37.13, 37.14, 37.15, 37.16, 37.17, 37.18, 37.19, 37.21, 37.22, 37.23, 37.24, 37.25, 37.26, 37.27, 37.28, 37.29, 37.31, 37.32, 37.33, 37.34, 37.4, 37.51, 37.61, 37.62, 37.64, 37.65, 37.80, 37.81, 37.82, 37.83, 37.94, 39.61, 39.99, 38.0, 38.1, 38.3, 38.40, 38.42, 38.43, 38.44, 38.45, 38.46, 38.47, 38.48, 38.49, 38.5, 38.6, 38.7, 38.8, 39.0, 39.1, 39.22, 39.23, 39.24, 39.27, 39.28, 39.29, 39.25, 39.26, 39.30, 39.31, 39.32, 39.41, 39.42, 39.43, 39.49, 39.50, 39.51, 39.52, 39.53, 39.55, 39.56, 39.57, 39.58, 39.59, 39.71, 39.72, 39.79, 39.8, 39.90, 39.91, 39.98

Hip/knee surgeries	CPT	27090, 27091, 27125, 27120, 27130, 27132, 27134, 27137, 27138, 27299, 27310, 27437, 27438, 27440, 27441, 27442, 27443, 27445, 27446, 27447, 27486, 27487, 27488, 29850, 29851, 29870, 29871, 29873, 29874, 29875, 29876, 29877, 29880, 29881, 29882, 29884, 29885, 29886, 29887, 29888
	ICD9A	77.66, 77.70, 77.76, 78.06, 78.16, 78.17, 78.46, 78.56, 79.26, 79.36, 80.05, 80.26, 80.46, 80.6, 80.76, 80.96, 81.47, 81.51, 81.52, 81.53, 81.54, 81.55

Digestive	CPT	42410, 42415, 43030, 43280, 43289, 43310, 43320, 43610, 43621, 43632, 43644, 43653, 43659, 43846, 44005, 44055, 44120, 44121, 44130, 44139, 44141, 44145, 44147, 44200, 44201, 44238, 44602, 44603, 44604, 44620, 44850, 45110, 45111, 45112, 45170, 46020, 46040, 47560, 47612, 48140, 48150, 48520, 49000, 49002, 49020, 49085, 49255, 49320, 49321, 49323, 49329
	ICD9A	42.0, 42.1, 42.19, 42.41, 42.42, 42.5, 42.6, 42.7, 42.81, 42.82, 42.83, 42.84, 42.85, 42.89, 42.91, 42.92, 42.99, 43.0, 43.5, 43.6, 43.7, 43.8, 43.9, 44.0, 44.21, 44.29, 44.40, 44.41, 44.42, 44.43, 44.44, 44.45, 44.46, 44.47, 44.48, 44.49, 44.5, 44.60, 44.61, 44.62, 44.63, 44.64, 44.65, 44.66, 44.67, 44.68, 44.69, 44.90, 44.91, 44.92, 44.93, 44.94, 44.95, 44.96, 44.97, 44.98, 44.99, 45.01, 45.02, 45.03, 45.10, 45.13, 45.15, 45.19, 45.26, 45.31, 45.41, 45.43, 45.49, 45.50, 45.51, 45.52, 45.61, 45.62, 45.63, 45.90, 45.91, 45.92, 45.93, 45.94, 45.95, 46.01, 46.02, 46.03, 46.1, 46.21, 46.22, 46.23, 46.24, 46.30, 46.31, 46.32, 46.33, 46.39, 46.40, 46.41, 46.42, 46.43, 46.44, 46.45, 46.46, 46.47, 46.48, 46.49, 46.51, 46.52, 46.6, 46.64, 46.71, 46.73, 46.74, 46.75, 46.76, 46.79, 46.81, 46.82, 46.85, 46.93, 46.94, 46.99, 47.93, 47.99, 48.0, 48.1, 48.4, 48.60, 48.66, 48.67, 48.68, 48.70, 48.71, 48.72, 48.73, 48.74, 48.75, 48.76, 48.77, 48.78, 48.79, 48.81, 48.82, 48.9, 48.93, 48.99, 51.00, 51.01, 51.02, 51.03, 51.04, 51.05, 51.06, 51.07, 51.08, 51.09, 51.10, 51.11, 51.12, 51.13, 51.14, 51.16, 51.17, 51.18, 51.19, 51.21, 51.32, 51.36, 51.37, 51.39, 51.40, 51.41, 51.42, 51.43, 51.44, 51.45, 51.46, 51.47, 51.48, 51.49, 51.51, 51.59, 51.60, 51.61, 51.62, 51.63, 51.64, 51.65, 51.66, 51.67, 51.68, 51.69, 51.7, 51.80, 51.81, 51.82, 51.83, 51.84, 51.85, 51.86, 51.87, 51.88, 51.89, 51.90, 51.91, 51.92, 51.93, 51.94, 51.95, 51.96, 51.98, 52.00, 52.01, 52.02, 52.03, 52.04, 52.05, 52.06, 52.07, 52.08, 52.09, 52.10, 52.12, 52.15, 52.16, 52.17, 52.18, 52.22, 52.11, 52.3, 52.4, 52.5, 52.6, 52.7, 52.8, 52.90, 52.91, 52.92, 52.93, 52.94, 52.95, 52.96, 52.97, 52.98, 52.99, 54.0, 54.1, 54.3, 54.4, 54.5, 54.6, 54.7

**Table 2:** Characteristics of VHA patients (*N* = 317,072).

Characteristic	All patients		White patients		African-American patients		Hispanic patients	
	Mean	SD	Mean	SD	Mean	SD	Mean	SD
Age in FY2005 (range 18–103)	62.7	14.9	64.2	14.6	55.7	14.4	58.1	16.4
Selim index of chronic physical conditions (range 0–15)	2.4	1.9	2.4	1.9	2.2	1.9	2.1	1.8
Charlson comorbidity score (range 0–23)	1.1	1.6	1.1	1.6	1.1	1.8	1.1	1.7

	*N*	%	*N*	%	*N*	%	*N*	%

White	254,761	80.4					11,793	70.1
African-American	54,307	17.1					4,380	26.0
Females	16,821	5.3	11,793	4.6	4,380	8.1		
Women	16,375	5.2	11,093	4.4	4,778	8.8	842	5.0
Males	300,697	94.8	243,668	95.7	49,529	91.2	15,979	94.99
Priority 1—50–100% disability, service-connected	53,826	17.0	41,165	16.2	10,676	19.7	3,141	18.7
Priority 2—30–40% disability, service-connected	21,572	6.8	16,220	6.4	4,624	8.5	1,186	7.1
Priority 3—10–20% disability, service-connected	34,928	11.0	27,672	10.9	6,290	11.6	1,859	11.1
Priority 4—catastrophically disabled	13,472	4.3	9,872	3.9	3,329	6.1	869	5.2
Priority 5—low income	112,482	35.5	88,290	34.7	21,739	40.0	7,192	42.8
Priority 6—special military service groups	9,644	3.0	7,907	3.1	1,465	2.7	784	4.7
Priority 7 or 8—other veterans, with copayments	71,148	22.4	63,635	25.0	6,184	11.4	1,790	10.7
Major depressive disorder	27,296	8.6	21,682	8.5	4,936	9.1	1,693	10.1
Hypertension	181,765	57.3	146,634	57.6	30,880	56.9	8,616	51.2
Dyslipidemia	158,207	49.9	134,831	52.9	19,689	36.3	7,386	43.9
Digestive surgeries	3,755	1.2	2,866	1.1	733	1.4	239	1.4
Hip/Knee surgeries	3,109	1.0	2,392	0.9	554	1.0	132	0.8
Vascular operations	11,330	3.6	9,113	3.6	1,799	3.3	506	3.0
CABG (coronary artery bypass graft)	1,899	0.60	1,614	0.6	203	0.4	87	0.5

**Table 3:** Distribution of selim chronic comorbid conditions for major minority race and ethnicity groups.

	White patients (*N* = 254,761)	Hispanic veterans (*n* = 16,794)	African-American veterans (*n* = 54,118)
Hypertension	57.2%	51.2%	56.8%
Diabetes	24.6%	29.6%	25.3%
Irregular heartbeat	9.8%	5.0%	5.1%
Peripheral vascular disease	5.2%	4.1%	3.9%
Heart attack	2.6%	2.0%	1.5%
Angina pectoris	1.9%	2.3%	1.4%
Transient ischemic attacks	1.2%	0.8%	0.7%
Stroke	3.6%	3.6%	4.2%
Congestive heart failure	5.4%	3.5%	4.4%
Chronic obstructive pulmonary disorder	14.7%	8.5%	10.0%
Hepatitis	3.4%	5.9%	7.9%
Anemia	7.4%	7.4%	10.1%
Cancer	11.4%	8.6%	9.5%
Skin cancer	2.5%	0.8%	0.2%
Diverticulitis	3.3%	3.3%	2.1%
Inflammatory bowel disease	0.7%	0.4%	0.3%
Gall bladder disease	0.9%	1.3%	0.8%
Gout	3.5%	2.2%	4.3%
Peptic ulcer	1.3%	1.5%	1.4%
Rheumatoid arthritis	1.1%	0.7%	0.7%
Osteoarthritis	17.4%	14.5%	16.3%
Hip arthropathies	1.4%	1.1%	1.4%
Other arthropathies	4.5%	4.0%	5.1%
Low-back pain	17.9%	20.6%	20.6%
Cataracts	12.6%	12.4%	11.1%
Seizures	0.4%	0.5%	0.6%
Thyroid disorder	7.2%	6.1%	3.6%
Urinary tract infection	2.9%	3.8%	4.0%
Enlarged prostate	12.2%	9.8%	7.9%
Prostatitis	0.8%	1.0%	0.9%

**Table 4:** Odds ratios associated with race and ethnicity indicators for patients undergoing surgery in the VHA (*N* = 317,072).

Dependent variable		
Effect	Odds ratio	95% confidence interval
*Digestive surgery*		
Depressed*	0.46	0.40–0.54
Hispanic*	1.26	1.10–1.43
African-American*	1.14	1.05–1.24
Age	1.00	1.00-1.00
Female*	1.30	1.12–1.52
Priority 1 (no copay)*	1.24	1.15–1.35
Selim physical comorbidity*	1.25	1.22–1.27
Charlson comorbidity score*	1.18	1.16–1.20

*Hip/Knee Surgery*		
Depressed*	0.49	0.42–0.57
Hispanic	0.84	0.71–1.00
African-American	1.04	0.95–1.15
Age*	0.99	0.99-0.99
Female	1.00	0.85–1.18
Priority 1 (no copay)*	1.77	1.63–1.91
Selim physical comorbidity*	1.65	1.62–1.69
Charlson comorbidity score*	0.68	0.66–0.70

*CABG*		
Depressed*	0.40	0.32–0.50
Hispanic	0.91	0.74–1.13
African-American*	0.56	0.49–0.65
Age*	0.98	0.98-0.99
Female*	0.29	0.20–0.43
Priority 1 (no copay)	1.06	0.94–1.19
Selim physical comorbidity*	1.44	1.40–1.48
Charlson comorbidity score	0.97	0.94–1.00

*Vascular operations*		
Depressed*	0.47	0.43–0.51
Hispanic*	0.88	0.81–0.97
African-American*	0.93	0.88–0.98
Age	0.99	0.99–1.00
Female*	0.51	0.44–0.58
Priority 1 (no copay)*	1.16	1.10–1.22
Selim physical comorbidity*	1.42	1.40–1.43
Charlson comorbidity score*	1.09	1.08–1.10

*95% confidence level excludes 1.0.
